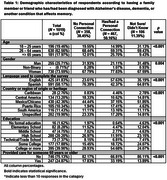# Comparative Analysis of Dementia Knowledge Among Latinos with Family or Friends Diagnosed with Memory‐Affecting Conditions: A cross‐sectional Study

**DOI:** 10.1002/alz70860_097846

**Published:** 2025-12-23

**Authors:** Maria C Mora Pinzon, Yelba Castellon‐Lopez, Susie Fernandez De Cordova, Maria del Carmen Rosales, Javier Neira Salazar, George Levy, Valentina B Flores Diaz, Diana C Martinez Garcia, Philip Sayegh

**Affiliations:** ^1^ Wisconsin Alzheimer's Institute, University of Wisconsin ‐ Madison, Madison, WI, USA; ^2^ Department of Medicine, Division of Geriatrics, School of Medicine and Public Health, University of Wisconsin‐Madison, Madison, WI, USA; ^3^ Cedars‐Sinai Medical Center, West Hollywood, CA, USA; ^4^ University of Wisconsin ‐ Madison, School of Medicine and Public Health, Madison, WI, USA; ^5^ University of Wisconsin School of Medicine and Public Health, Madison, WI, USA; ^6^ School of Medicine and Public Health, University of Wisconsin ‐ Madison, Madison, WI, USA; ^7^ University of Wisconsin ‐ Madison, Madison, WI, USA; ^8^ University of California, Los Angeles, Los Angeles, CA, USA

## Abstract

**Background:**

Latinos/as/es/xs may face significant disparities in accessing diagnostic healthcare services for memory‐related conditions, impeding identification of underlying etiologies, such as Alzheimer's disease and related dementias (ADRD). Our study explored if having friends or family members with a memory‐affecting condition influenced Latine perspectives on these cognitive impairments. The goal was to improve our understanding of knowledge disparities that might exist, which is crucial for the effective development of interventions and support strategies.

**Methods:**

A validated survey was distributed through multiple channels in the United States and Puerto Rico, including online platforms, in‐person through promotoras, and mailings to households between September 2021 and April 2024. Study participants were self‐identified Latino/a/e/x adults aged 18 or older. The 47‐item survey used a 5‐point Likert scale to assess various aspects of dementia knowledge, including the importance of seeking medical care, perceived embarrassment, and beliefs about the efficacy of care provided by doctors. Descriptive statistics were used to summarize data, and Chi‐square and other nonparametric tests were employed to compare differences between groups (with/without a personal connection to a memory affecting condition).

**Results:**

The survey was completed by 1,019 Latino/a/e/x individuals, 74% of whom were women, with an average age of 46.2 years (range 18–94). Half of the respondents had a family member or friend diagnosed with ADRD or other memory‐affecting condition, and they demonstrated higher knowledge about dementia (*p* <0.001). When asked about their perspectives if they themselves were to develop memory problems that affected daily life, they reported being more likely to consider seeking medical care, less embarrassment, and being less likely to believe that doctors could provide training for family members or useful medications (*p* = 0.01). Among all respondents, 75.8% reported believing it was very/extremely important to care for people at home, with no significant difference according to personal connection.

**Conclusions:**

Latinos/as/es/xs with a personal connection to memory‐affecting conditions were less likely to believe that doctors could provide useful medications or training for families. These findings underscore the need for targeted educational interventions and improving access to high‐quality healthcare services to ensure timely diag nosis and effective care for those affected by ADRD within the Latino/a/e/x community.